# Research on privacy protection in the context of healthcare data based on knowledge map

**DOI:** 10.1097/MD.0000000000039370

**Published:** 2024-08-16

**Authors:** Ting Ouyang, Jianhua Yang, Zongyun Gu, Lei Zhang, Dan Wang, Yuanmao Wang, Yinfeng Yang

**Affiliations:** a School of Medical Informatics Engineering, Anhui University of Chinese Medicine, Hefei, China; b Anhui Computer Application Research Institute of Chinese Medicine, China Academy of Chinese Medical Sciences, Hefei, China.

**Keywords:** CiteSpace, healthcare data, knowledge maps, privacy protection

## Abstract

With the rapid development of emerging information technologies such as artificial intelligence, cloud computing, and the Internet of Things, the world has entered the era of big data. In the face of growing medical big data, research on the privacy protection of personal information has attracted more and more attention, but few studies have analyzed and forecasted the research hotspots and future development trends on the privacy protection. Presently, to systematically and comprehensively summarize the relevant privacy protection literature in the context of big healthcare data, a bibliometric analysis was conducted to clarify the spatial and temporal distribution and research hotspots of privacy protection using the information visualization software CiteSpace. The literature papers related to privacy protection in the Web of Science were collected from 2012 to 2023. Through analysis of the time, author and countries distribution of relevant publications, we found that after 2013, research on the privacy protection has received increasing attention and the core institution of privacy protection research is the university, but the countries show weak cooperation. Additionally, keywords like privacy, big data, internet, challenge, care, and information have high centralities and frequency, indicating the research hotspots and research trends in the field of the privacy protection. All the findings will provide a comprehensive privacy protection research knowledge structure for scholars in the field of privacy protection research under the background of health big data, which can help them quickly grasp the research hotspots and choose future research projects.

## 1. Introduction

The application of emerging information technologies, such as artificial intelligence (AI), big data analytics, and the Internet of Things (IoT), has revolutionized the healthcare sector, offering unprecedented opportunities for medical data collection, storage, and analysis.^[1]^ However, this rapid digitization of healthcare services has introduced profound challenges to the privacy and security of medical data. For example, the interconnected nature of medical devices and IoT-enabled sensors has increased the risk of cyber threats and unauthorized access to patient data.^[2]^ Additionally, the integration of digital health tools such as mobile apps and telemedicine platforms has blurred the boundaries between traditional healthcare settings and digital platforms, presenting new challenges for patient privacy.^[3]^ Accordingly, the concern of people about the security and confidentiality of patient data is also increasing.^[4,5]^ Therefore, it is of great practical significance to study privacy protection in the context of medical big data. Strengthening privacy protection of medical data not only safeguards individual rights and data security, but also facilitates the reasonable utilization and sharing of medical information, driving the development and innovation of the healthcare industry.

In this context, researchers have proposed various ideas and conducted research on privacy protection. For instance, Lin and Sun et al addressed patient privacy protection in intelligent wearable devices and machine learning, employing differential privacy technology.^[6,7]^ Bauer et al conducted a security analysis of heterogeneous medical data using the Integrated Data Repository Toolkit.^[8]^ In addition, Zhang et al developed a privacy-optimized clinical path query scheme to establish a secure path query in a medical cloud server.^[9]^ Furthermore, Hussien and Huang et al addressed medical privacy protection using blockchain technology.^[10,11]^ Mittelstadt and Salerno et al explored the ethical challenges and opportunities of privacy protection in health big data.^[12,13]^ Additionally, discussions have extended to laws, regulations on gene data, health information, and regional data sharing.^[14–20]^

However, there lacks a comprehensive overview of global research on privacy protection. Few studies delve into the comprehensive knowledge structure and research hotspots of privacy protection from a bibliometric perspective. CiteSpace, a knowledge mapping visualization tool based on scientific bibliometrics, is frequently employed to analyze research trends and changes in various fields.^[21]^ Compared to other literature visualization software, CiteSpace offers a user-friendly interface, powerful functions, and easy expandability.^[22]^ It also provides researchers with a range of functionalities to visualize and analyze literature data, revealing connections and trends among publications. In addition, through generating keyword co-occurrence networks, citation networks, and spatiotemporal evolution maps, CiteSpace excels in scientific visualization and knowledge discovery. These features aid researchers in understanding literature data, identifying new research directions, and pinpointing hot topics.^[23]^ Employing CiteSpace for information visualization of privacy protection research in the context of medical big data can provide valuable insights for medical data security research.

Presently, a bibliometric analysis was constructed based on the privacy protection and healthcare data related studies retrieved from the Web of Science (WOS) database from 2012 to 2023. Using the knowledge map tool CiteSpace, the current research status, hot spots, and the future trends with regards to privacy protection was analyzed from the macro-level to the micro level. First, t Analysis of countries/regions provides insight into their important role, and keyword analysis reveals current hot spots and future directions. Finally, the research results are discussed to understand the current status of privacy protection research and predict future trends. All the results offer valuable references for scholars in knowledge mapping and visual analysis, as well as those delving into big data research. They alleviate the workload of relevant researchers and provide guidance for understanding this field. The results offer essential knowledge support for domestic and foreign researchers in related fields.

## 2. Materials and methods

### 2.1. Data sources

All data for this study was extracted from the SCI-EXPANDED, SSCI, and A&HCI databases within the WOS Core Collection. Due to the authority and high quality of these databases, the data from WOS are often utilized as a key source for analyzing research trends and frontiers within academic disciplines. Using the search formula of TS= “health big data” AND “privacy protect*” OR TS= “health big data” AND “Privacy security” OR TS= “health big data” AND “privacy concern*,” the relevant literatures from January 1, 2012 to December 31, 2023 in WOS were retrieved.

### 2.2. Eligibility and exclusion criteria

**Figure 1.**
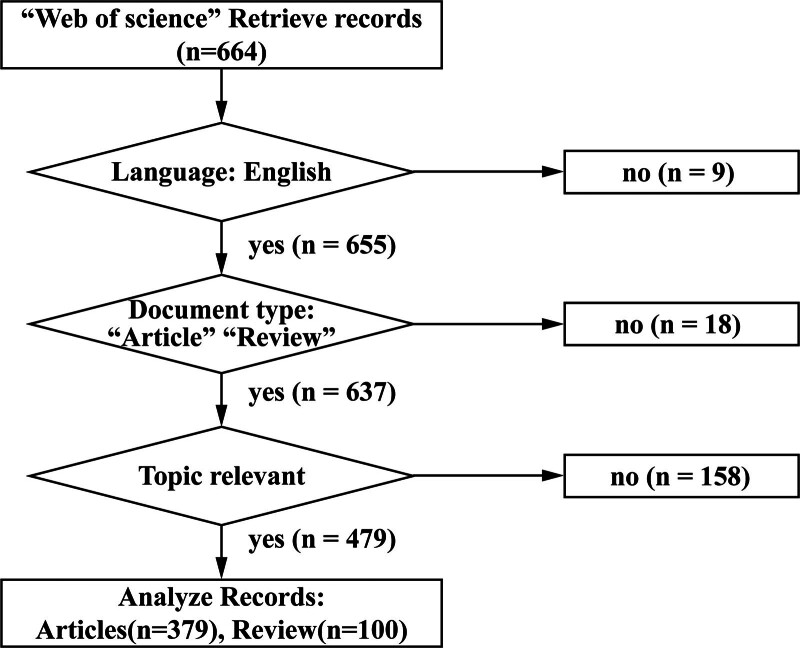
Literature retrieval and exclusion strategies.

The inclusion and exclusion criteria selected for this study are outlined as follows: (1) the language of the articles was limited to “English.” (2) The type of article was selected as “Article” and “Review Article,” which are involved specific keywords related to the privacy protection and privacy concern in the context of health big data between 2012 and 2023. Additionally, the exclusion criteria are as listed as follows: (1) letters to the editor and unpublished data; (2) articles containing missing key information or deemed irrelevant by multiple researchers after thorough reading and discussion were excluded from the study. Moreover, studies with conference abstracts, press releases, litigation documents, editorial materials, and similar content were not considered for inclusion. Employing these criteria, a total of 479 relevant publications were obtained from the WOS and used for further analysis. Figure 1 depicts the detailed process of literature search and exclusion strategy.

### 2.3. Data processing analysis

To elucidate the current research landscape, identify hotspots, and uncover emerging trends within the domain of privacy protection in health big data, the visualization software CiteSpace was utilized in the present work.^[24]^ As one of the foremost tools for visualizing knowledge graphs, CiteSpace primarily employs co-citation analysis and pathfinding network algorithms to compute specific domain literature, thereby exploring key evolutionary paths and knowledge turning points within a given discipline.^[25]^ In CiteSpace, the research frontier is delineated based on the identification of pivotal terms extracted from topics, abstracts, descriptors, and document identifiers. Herein, the analysis focuses on various factors including keywords, authors, and countries/regions. When using Citespace analysis, the parameter Timeslicing was set to from 2012 to 2023 years. The perslice was set to 1 year and the scope option is set to Winthin Slice, and the TopNperslice N is set to 50. To simplify the plots and avoid the complexity of the content, we examined the options of “pruning sliced networks,” “pruning the merged network,” and “path-finder,” and the remaining parameters were kept at their default values.

### 2.4. Statistical methods

Presently, a dataset consisting of 479 documents retrieved from the WOS was exported in text format. During exportation, the “full records and cited references” were selected to ensure comprehensive inclusion of bibliographic details such as author, title, journal, keywords, abstract, publication year, and citation count. Subsequently, the dataset was imported into the CiteSpace software to construct the co-occurrence and cluster analysis of authors, research institutions, countries, funding sources, disciplinary attributes, and keywords. All the results are presented in the form of a knowledge graph to visually represent the relationships and patterns within the research landscape.

### 2.5. Ethical statement

Because we did not perform the animal experiments in the present study, there is no Ethical state in the Methods section.

## 3. Results

### 3.1. Time distribution of publications

**Figure 2.**
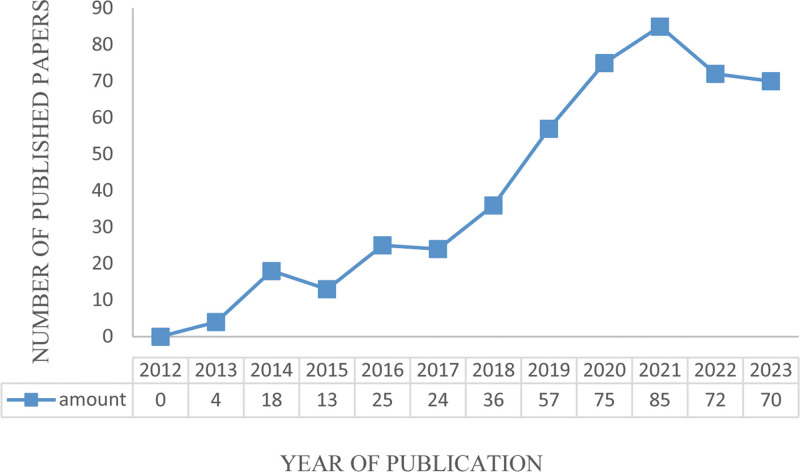
Annual publication trend graph.

By analyzing the publication trends over time, one can discern the evolving research enthusiasm, scale, and pace of development within this field.^[26]^ Currently, the annual publication count of the 479 included articles was summarized in a line chart illustrating the yearly publication volume. It is evident that privacy protection, as an important domain derived from the application of big data technology, has garnered increasing attention, as evidenced by a rising number of publications (Fig. 2).

In addition, it can be seen from Figure 2 that in 2012, there were no publications since countries began to launch their big data construction plans. Subsequently, from 2013 onwards, the number of publications related to privacy protection in the context of health big data has generally shown an upward trend, which can be roughly divided into 2 stages. The first stage (2013–2017) is a period of steady growth, and the annual volume of publications grows steadily, while the second stage (2018–2021) is a period of rapid growth, and the curve of publications rises linearly, reaching 85 in 2021. Obviously, there are 70 articles published in 2023, indicating that with the deepening of the application of big data technology in the health field, research in the field of privacy protection is in a period of rapid development.

### 3.2. Author and citation rate analysis of publications

**Figure 3.**
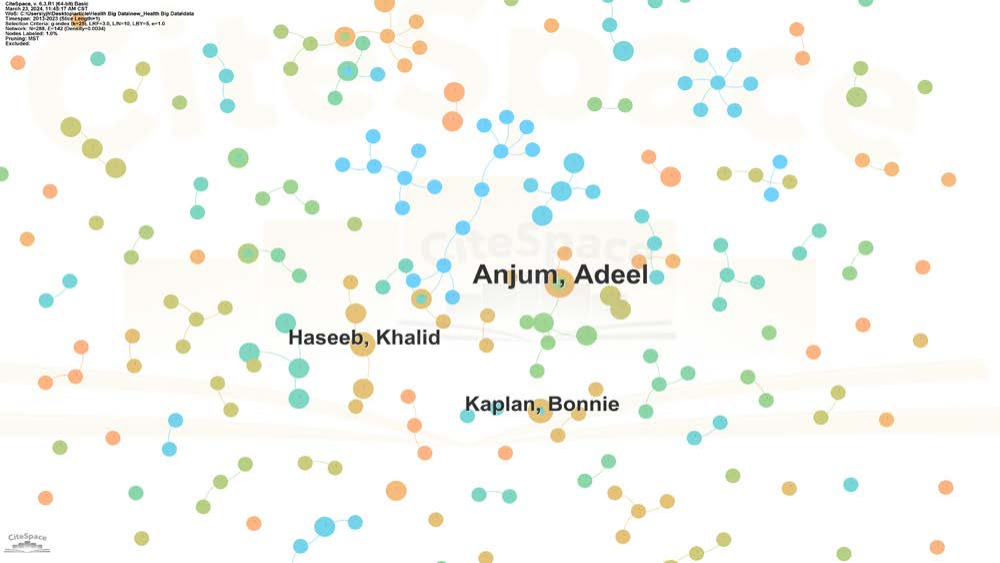
Author cooperation network diagram.

The volume of published papers can serve as an indicator of an author’s scientific contribution. Therefore, conducting a quantitative analysis of authors provides insights into identifying core and prolific authors within a specific field.^[27]^ As depicted in Figure 3, each node represents an author, with the size of the node reflecting the frequency of the author’s appearance in the research field. Links between authors signify collaborative relationships.^[25]^ Notably, Anjum, Adeel emerges as the most prolific author with 5 articles published, indicating collaborative ties with other authors. However, the visualization suggests that the collaboration network among authors appears relatively simplistic and loosely connected.

Additionally, the citation count of papers not only indicates the contribution of authors, but also highlights the developmental trajectory and focal areas of the research field.^[28]^ Consequently, an analysis of the number published papers based on their total citation frequency was carried out in the current work and the corresponding results are summarized in Table 1. Clearly, the paper wrote by Islam SMR entitled “The Internet of Things for Health Care: A Comprehensive Survey” has the highest citation rate, with a total number of 1335, an average annual citation rate of 133.5, and the paper of Hsieh CY ranked the second citation rate with 675. In addition, the paper entitled “Big Data In Health Care: Using Analytics To Identify And Manage High-Risk And High-Cost Patients.” Has the third citation rate. All the results show that the research on privacy protection of big data applications in medicine, AI and IoT technology is a hot topic.

**Table 1:** Scholars with a high frequency of citations and their related information.

No.	Paper title	Author	Average annual Citation frequency	Total citation
1	The internet of things for health care: A comprehensive survey	Islam SMR	133.5	1335
2	Taiwan’s National Health Insurance Research Database: Past and future	Hsieh CY	112.5	675
3	Big data in health care: Using analytics to identify and manage high-risk and high-cost patients	Bates DW	53.82	592
4	Internet of things security: A survey	Alaba FA	67.38	539
5	The quantified self: Fundamental disruption in big data science and biological discovery	Swan M	41.75	501
6	Privacy in the age of medical big data	Price WN	71.67	430
7	Big data for health	Andreu-perez J	38.5	385
8	Federated learning of predictive models from federated Electronic Health Records	Brisimi TS	52.86	370
9	Differential privacy techniques for cyber physical systems: A survey	Hassan MU	60.6	303
10	The ethics of big data: Current and foreseeable issues in biomedical contexts	Mittelstadt BD	32.89	296

### 3.3. Analysis of national distribution and cooperation network

Through statistical analysis of countries and research institutions in the field of privacy protection research in the field of health big data, authoritative scholars and experts in this field can be obtained.^[27]^ Currently, a total of 479 articles from 76 different countries around the world were analyzed according to the frequency of publication. Table 2 depicts the relevant information of top 10 countries with the most publications in the world for privacy protection research in the field of health big data. Obviously, the USA and CHINA together contribute more than half of the literature output, indicating that they have played an excellent role in privacy protection in health big data research. It is worth noting that although the number of scientific literature published by AUSTRALIA is lower than that of CHINA, the average number of citations per article is higher than that of CHINA, accounting for 38.66 and ranking the second highest average citation rate. Similarly, the number of documents in ENGLAND, SWITZERLAND, and CANADA is lower than that of CHINA, the average number of citations per article is higher than that of CHINA, showing that the impact of these researches in the field of privacy protection is also important.

**Table 2:** Top 10 countries with the most publications in the world for privacy protection research in the field of health big data.

No.	Countries/Regions	Record count	Constituent ratio (%)	Total citations	Citations Per article
1	USA	144	30.063	8399	63.14
2	CHINA	105	21.921	2954	29.7
3	ENGLAND	72	15.031	2577	37.61
4	AUSTRALIA	44	9.186	1647	38.66
5	INDIA	38	7.933	817	22.13
6	GERMANY	28	5.846	784	28.25
7	SWITZERLAND	27	5.637	865	33.11
8	CANADA	26	5.428	834	33.12
9	NETHERLANDS	26	5.428	722	28.58
10	SPAIN	23	4.802	423	18.52

**Figure 4.**
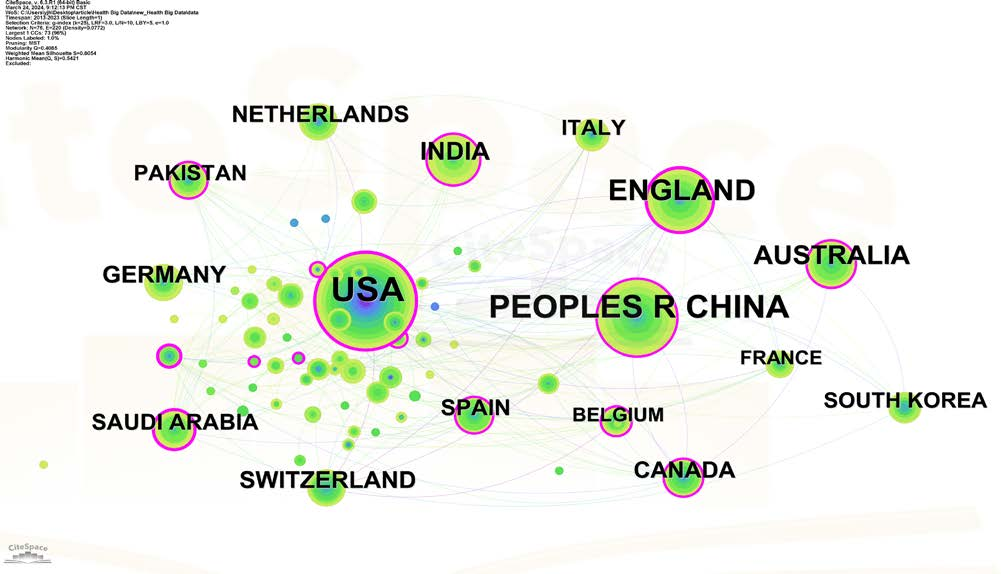
Country cooperation network diagram.

Furthermore, the distribution of knowledge ownership and collaboration networks within medical health data privacy protection research across various countries is also examined by CiteSpace. Figure 4 illustrates the national cooperation network with a display threshold set at Recs ≥ 10 for each country. In this net, circle size corresponds to the volume of literature, while different colors indicate publication years. The analysis encompasses 79 countries and identifies 264 collaborative links between them. Evidently, the USA occupies a central position within the cooperation network, boasting the highest publication count and fostering close collaborative ties with other nations. Similarly, CHINA ranks second in publication volume, maintaining cooperative relationships with various countries. Moreover, the figure also illustrates a relatively loose inter-country or inter-regional cooperation framework in the domain of health big data privacy protection research. Consequently, there is a compelling need to enhance international cooperation in this research field, thereby amplifying both the depth and breadth of scholarly investigation.

### 3.4. Analysis of research institutions distribution

Generally, a crucial indicator for assessing a country’s research strength lies in the presence of academic institutions and esteemed scholars involved in pioneering investigations within the discipline. To evaluate institutional cooperation in the field of privacy protection, the top 10 research institutions in the privacy protection research field of health big data were identified based on the publication volume, which is summarized in Table 3. The results show that universities serve as the core institution of privacy protection research. Among them, Harvard University and the University of London jointly hold the top position with 18 published articles each, followed by the University of California System. Notably, the institutions that have published more than 10 scientific papers are mainly from the top universities or branches in the USA and the ENGLAND.

**Table 3:** Top 10 research institutions in the world.

No.	Organization name	Countries/Regions	Record count (piece)	Composition Ratio (%)
1	Harvard University	USA	18	3.76
2	University of London	ENGLAND	18	3.76
3	University of California System	USA	14	2.92
4	University of Oxford	ENGLAND	12	2.51
5	University College London	ENGLAND	11	2.30
6	Imperial College London	ENGLAND	9	1.88
7	Stanford University	USA	9	1.88
8	University of Texas System	USA	9	1.88
9	Utrecht University	HOLLAND	9	1.88
10	Utrecht University Medical Center	HOLLAND	9	1.88

### 3.5. Visual map analysis of research hotspots

**Figure 5.**
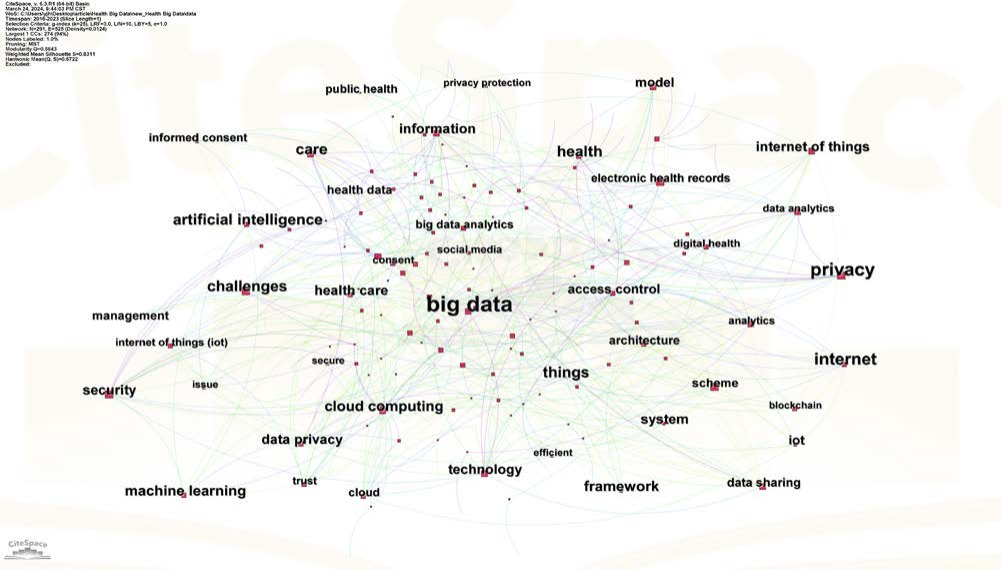
Heat map of privacy protection research in the field of health big data.

**Figure 6.**
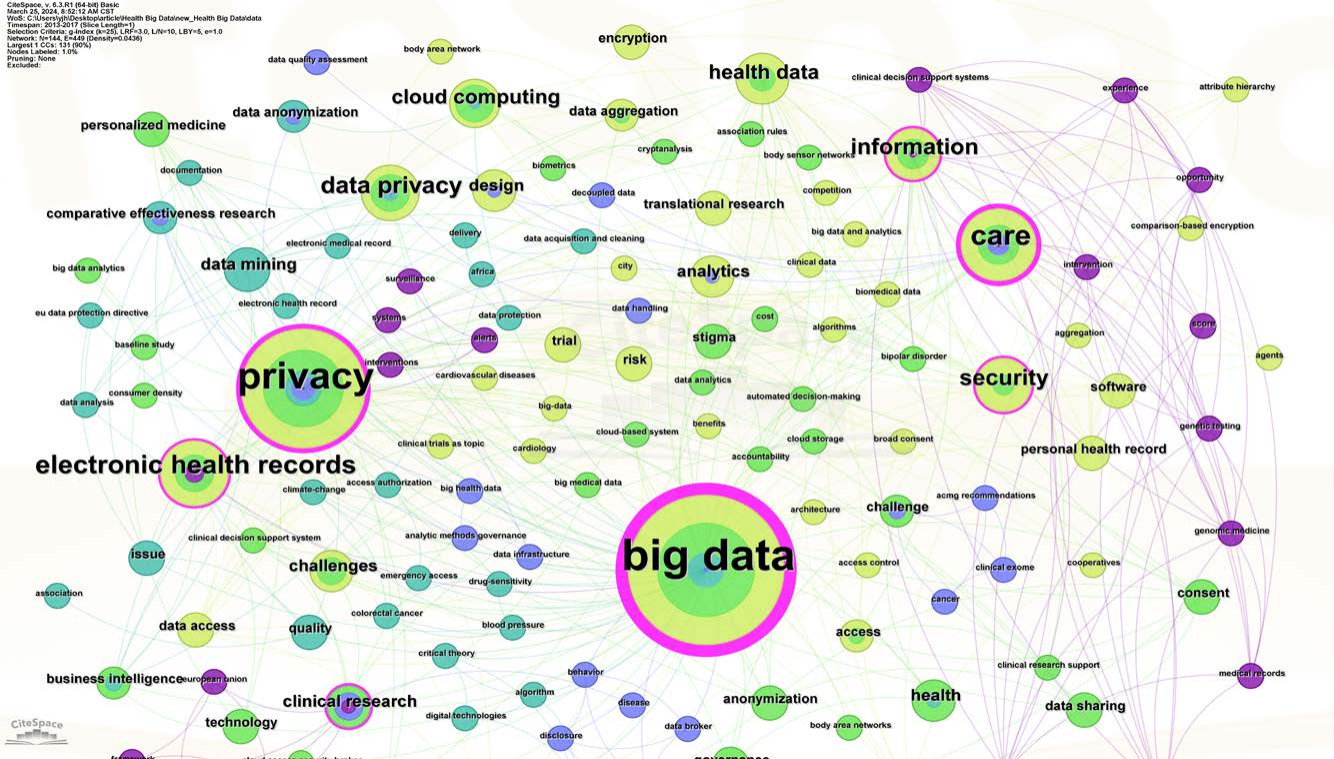
Density map of research hotspots (2013–2017).

**Figure 7.**
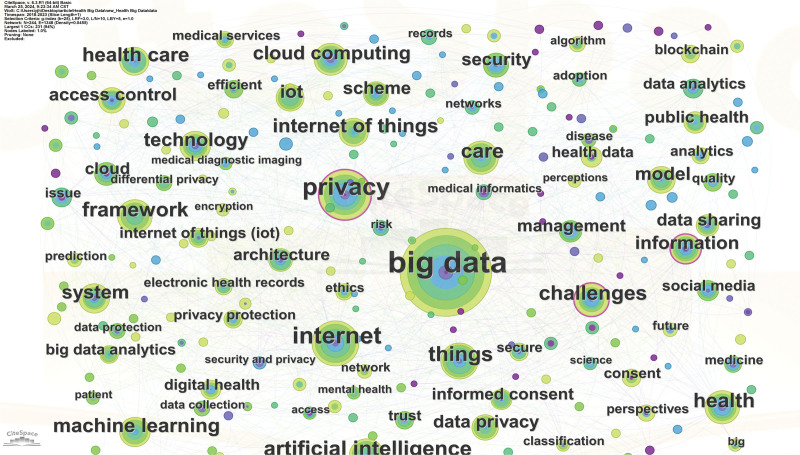
Density map of research hotspots (2018–2023).

In general, co-word analysis, as one of the most important contents of bibliometric analysis, is used to discover the connection between disciplines in a research field, extract the research hotspots in the field, and then further trace the development of science.^[28]^ Since the keywords can represent the core point of the document, presently, the keyword co-occurrence visual map analysis was carried out by the CiteSpace (Fig. 5). Through the keyword analysis, the research hotspots within a specific field was determined. To mitigate statistical inaccuracies that may influence the identification of research hotspots, it is imperative to initially eliminate generic terms synonymous with the research domain. Subsequently, akin and synonymous keywords should be amalgamated, followed by an analysis of the co-occurrence map of high-frequency keywords. Table 4 summarizes the top 20 high-frequency keywords from 479 articles and the corresponding co-word networks were depicted in Figures 6 and 7.

**Table 4:** High-frequency keywords for privacy protection research in the context of health big data.

No.	Keywords	Frequency	Centrality	No.	Keywords	Frequency	Centrality
1	Big data	222	0.16	11	Health care	31	0.07
2	Privacy	156	0.18	12	Artificial Intelligence	31	0.01
3	Internet	52	0.02	13	Blockchain	29	0.04
4	Security	50	0.12	14	Information	27	0.11
5	Challenge	41	0.14	15	System	27	0.03
6	Care	38	0.11	16	Iot	26	0.02
7	Health	37	0.03	17	Technology	26	0.03
8	Ethics	36	0.04	18	Cloud computing	26	0.04
9	Framework	34	0.04	19	Healthcare	24	0.04
10	Thing	33	0.02	20	Data privacy	23	0.04

As shown in Table 4, the keyword with the highest frequency is “Big data,” followed by the keyword “Privacy,” indicating the importance of these keywords and the research hotspots. In addition, another parameter, that is, the centrality, also implies the crucial role of these nodes. The larger the purple circle on the outer edge of the node on the cooperative network map (Figs. 6 and 7), the higher the centrality of the representative node, which means that the node is more important and is a research hotspot. Obviously, the keywords with the high frequency also have the high centrality. For example, the centrality of the keyword “Big data” with the highest frequency is 0.16, and the centrality of the keyword “Privacy” with the second highest frequency is the highest, reaching 0.18 (Table 4). Interestingly, the frequency of the keyword “Internet” is relatively high, but its centrality is very low, only 0.02 (Table 4). By contrast, the frequency of the keyword “Information” is 27, but the centrality is very high, reaching 0.11 (Table 4). Overall, a total of 6 important keywords have high centrality, indicating the research hotspots in the field of medical health data privacy protection research, which are also demonstrated in Figures 6 and 7.

In addition, careful analysis of Figures 6 and 7 shows the evolution of privacy protection research hotspots in the context of healthy big data. Specifically, before 2015, there were few studies on the privacy protection, which was consistent with the analysis results of Figure 2. However, from 2013 to 2017, the focus of research was mainly on the digitization of health information, how to effectively manage health information, and the ethical issues behind it. After 2017, with the in-depth application of new technologies in the field of health, privacy protection has once again become a research hotspot. At this time, the research focus is mainly on how new technologies protect privacy and what opportunities and challenges do new technologies bring to personal health information.

Besides, due to the outbreak of COVID-19 in 2020, the research on privacy issues in the field of public health has gradually increased. Figure 7 depicts the density map of research hotspots from 2018 to 2023. Clearly, privacy, big data, internet, challenge and information become the high-frequency keywords, reflecting the research hotspots and research trends in specific areas.

## 4. Discussion

In the current study, using the specific formula, a total of 479 documents retrieved from WOS are bibliometrically analyzed by the software of CiteSpace. The co-occurrence and cluster analysis of authors, research institutions, countries and keywords of publications are also carried out. Through building the knowledge graph of privacy protection in the context of healthy big data, the insights into the research hotspots, development history and trends of this research field are provided.

The results show that after 2013, with the deepening of the application of big data technology in the health and medical field, the amount of research related to privacy protection has gradually increased (Fig. 2). Compared with the research of larger data technology, the international cooperation and the cooperation between authors in the field of privacy protection are relatively less, and the closeness of cooperation is not high, and no more influential authors have been formed (Figs. 3 and 4). The reason for this weak cooperation may come from the interdisciplinary communication barriers between health care, computer science, and law disciplines. In addition, strict data privacy regulations hinder data sharing and the resource constraints such as capital and time also hinder cooperation. To address the issue of limited collaboration among authors, interdisciplinary collaboration platforms should be constructed, regulating privacy frameworks, promoting data sharing, and providing incentives for collaborative efforts. Additionally, education and training programs that focus on interdisciplinary collaboration and data privacy are essential for researchers to effectively engage in collaborative research.

Moreover, citation rate analysis of publications reveals that the research on privacy protection of big data applications in medicine, AI and IoT technology is a hot topic (Table 1). Actually, examination of the privacy protection in big data applications within medicine, AI and IoT is pivotal. With the in-depth application of research technology, privacy protection technologies in the fields of public health, cloud computing, and access control have gradually attracted the attention of researchers (Figs. 6 and 7), and increasing researchers have developed strong protection mechanisms. For example, more and more studies have proposed a comprehensive evaluation framework and model using fuzzy logic,^[29,30]^ multi-criteria decision analysis,^[31,32]^ neural network^[33–35]^ and analytic hierarchy process methods.^[36]^ Using these approaches, the security and persistence of software can be evaluated and optimized, and its ability to protect user data privacy in the process of processing can be indirectly enhanced.

Besides, visual map analysis of research hotspots demonstrate that 6 important keywords including privacy, big data, internet, challenge, care, and information have high centrality and frequency, reflecting the research hotspots and research trends in specific areas (Table 4 and Fig. 6). Among them, privacy and big data occupy the more crucial roles, indicating the importance of privacy protection in the context of health big data (Fig. 7). Indeed, digitization and extensive data collection have made the health information of individuals more vulnerable.^[37]^ Safeguarding this data is critical, as its misuse can compromise personal privacy, data security, and healthcare quality.^[38]^ Meanwhile, policymakers should enact regulations to protect public and individual privacy rights. In addition, research on personal information privacy protection is crucial across diverse cultural and legal contexts, shaping individual rights, societal values, and legal frameworks.^[39]^ These contexts influence attitudes toward data sharing, requiring culturally sensitive strategies. Consequently, the introduction of relevant laws and regulations on health data privacy protection and the establishment of specialized regulatory authorities can reduce the risk of privacy security issues in the process of medical and health big data processing.^[40–42]^

Although this study predicts future research hotspots in the field of privacy protection, there are limitations due to the use of 1 software for analysis. In addition, with the number of the publications increased, the accuracy of these predictions still needs to be continuously tested and verified. In addition, with the constant advancement of technology and societal changes, the domain of privacy protection may encounter new challenges and opportunities. Therefore, there needs to be further research into privacy protection in the context of big data.

## 5. Conclusions

Big data provides many advantages and potential for innovation in various fields, but it also brings many problems and challenges. In particular, the problems of data security and reliability in medical health big data have led to more and more attention to privacy protection research in this context. Therefore, the papers about the privacy protection in the context of healthcare originated from WOS database from 2012 to 2023 are bibliometrically analyzed employing the knowledge graph visualization software. The main findings are listed as follows.

1. After 2013, with the deepening of the application of big data technology in the health field, the number of research on the privacy protection is rapidly increased.
2. The analysis of the citation rate of the publications reveals that AI and IoT technology are hot topics in this field.
3. The universities serve as the core institution of privacy protection research, and USA and China have higher number of publications, but the cooperation between countries is relatively weak.
4. Six important keywords including privacy, big data, internet, challenge, care, and information have high centrality and frequency, showing the research hotspots and research trends in the field of the privacy protection.

In summary, all the results will provide insights into research trends, challenges, and the need to prioritize privacy protection in the context of healthcare data.

## Acknowledgments

The authors would like to thank the Major Scientific Research Project of Anhui Provincial Department of Education (Grant No. 2023AH040102), the Anhui Province quality projects (Grant No. 2022AH010038 and No. 2023sdxx027), the Key Humanities Projects of Anhui University of Traditional Chinese Medicine (Grant No. 2021rwzd12), the Middle-aged Young Teacher Training Action Project of Anhui Provincial Department of Education (Grant No. JNFX2023020) and the General Project of Teaching Research in Anhui Province (Grant No. 2023jyxm0370).

## Author contributions

**Conceptualization:** Dan Wang.

**Formal analysis:** Jianhua Yang.

**Investigation:** Jianhua Yang.

**Methodology:** Lei Zhang.

**Project administration:** Yuanmao Wang.

**Resources:** Zongyun Gu.

**Supervision:** Yinfeng Yang.

**Writing – original draft:** Ting Ouyang.

**Writing – review & editing:** Yinfeng Yang.
